# Water-catalyzed ultrafast formation of hydronium clusters

**DOI:** 10.1093/nsr/nwag443

**Published:** 2026-07-17

**Authors:** Menghang Shi, Chenxu Lu, Zhubin Hu, Wenbin Zhang, Jiaxuan Chen, Peifen Lu, Haitao Sun, Zhenrong Sun, Yang Tian, Jian Wu

**Affiliations:** State Key Laboratory of Precision Spectroscopy, East China Normal University, Shanghai 200241, China; State Key Laboratory of Precision Spectroscopy, East China Normal University, Shanghai 200241, China; State Key Laboratory of Precision Spectroscopy, East China Normal University, Shanghai 200241, China; State Key Laboratory of Precision Spectroscopy, East China Normal University, Shanghai 200241, China; State Key Laboratory of Precision Spectroscopy, East China Normal University, Shanghai 200241, China; State Key Laboratory of Precision Spectroscopy, East China Normal University, Shanghai 200241, China; State Key Laboratory of Precision Spectroscopy, East China Normal University, Shanghai 200241, China; State Key Laboratory of Precision Spectroscopy, East China Normal University, Shanghai 200241, China; Shanghai Key Laboratory of Green Chemistry and Chemical Processes, School of Chemistry and Molecular Engineering, East China Normal University, Shanghai 200241, China; State Key Laboratory of Precision Spectroscopy, East China Normal University, Shanghai 200241, China

**Keywords:** ultrafast dynamics, proton transfer, reaction microscopy, water catalysis, hydronium clusters

## Abstract

Water catalyzes proton transport through hydrogen-bond networks, yet the minimal structural complexity required to initiate this behavior remains unresolved. Here, we track the birth of the hydrated proton by monitoring the ultrafast formation of size-selected hydronium clusters (H_2_O)*_n_*_−__1_H^+^ in real time using femtosecond reaction microscopy. We observe a kinetic collapse in the formation timescale, which drops from ∼259 fs for the water dimer (*n* = 2) to ∼70 fs for the trimer (*n* = 3) before converging for larger sizes. This identifies the cyclic water trimer as the minimal catalytic unit. *Ab initio* molecular dynamics simulations reveal that this acceleration is not driven by the initial proton transfer step, which remains ultrafast, but by the elimination of the reaction barrier governing the separation of the hydronium-solvated products. Our findings demonstrate that the cooperative action of just three water molecules establishes the critical topological threshold for efficient water catalysis, providing a microscopic blueprint for the onset of broader aqueous reactivity.

## Teaser

Real-time observations of hydronium cluster formation uncover that a minimal cyclic trimer of water molecules effectively suppresses reaction barriers, providing a microscopic blueprint for the onset of catalytic efficiency.

## INTRODUCTION

Water is the indispensable medium for life and the vast majority of chemical processes [1–4]. Far from being a passive solvent, water acts as an active catalyst that drives critical transformations, from enzymatic activity [5,6] and protein folding [7–9] to acid–base neutralization [10,11]. The fundamental physical basis for this catalytic power lies in the rapid transport of protons through the hydrogen-bond network, often described by the Grotthuss mechanism [12,13]. Central to this process are the protonated water clusters or hydronium clusters. These species are not merely transient reaction byproducts, but also the distinct functional units that stabilize and convey positive charge through the aqueous environment [14,15]. Consequently, a molecular-level understanding of water catalysis requires more than knowledge of their equilibrium structure, it demands elucidation of the dynamical principles governing their ultrafast formation. In particular, revealing how a neutral water network reorganizes to facilitate the birth of a hydronium ion is essential for uncovering the microscopic origins of aqueous reactivity.

However, studying this formation process presents a formidable challenge. Recent breakthrough experiments utilizing liquid-phase ultrafast electron diffraction have successfully captured the short-lived OH(H_3_O^+^) radical-cation intermediate and its subsequent dissociation in bulk liquid water [16]. While these liquid-phase studies reveal the macroscopic geometric evolution and collective structural reorganization of the solvent network, the immense complexity of the bulk environment obscures the specific, foundational structural motifs fundamentally responsible for the catalysis. Consequently, it remains difficult to identify the minimal unit required to switch on this function. In contrast, gas-phase studies of small clusters have previously focused on static spectroscopy [17–20] or intrinsic proton transfer dynamics in isolated dimers [21,22], often missing the broader collective dynamics that emerge with increasing cluster size. While the intrinsic photodissociation of isolated water monomers is well characterized [23], the presence of even a limited hydrogen-bond network fundamentally alters the reaction pathways, such as driving complex proton relays in water nanodroplets [24]. A critical knowledge gap remains in the transition regime: How does the addition of a single water molecule alter the energy landscape of the reaction? Does catalytic efficiency evolve smoothly with cluster size, or does it arise sharply at a distinct structural threshold where the water network acquires the ability to stabilize charge? Addressing these questions requires tracking reaction dynamics in real time with molecular-level resolution. An ideal model system is the photoionization-induced reaction: (H_2_O)*_n_* + *n*ħω → (H_2_O)*_n_*_-1_···H_2_O^+^ + e^−^ → (H_2_O)*_n_*_−__1_H^+^···OH → (H_2_O)*_n_*_−__1_H^+^ + OH [21,22,25], which provides a well-defined platform for investigating proton transfer and solvation dynamics. By measuring the formation time of the resulting hydronium clusters, we can quantitatively assess the catalytic influence of the hydrogen-bond network in small clusters.

Here, we employed femtosecond reaction microscopy to real-time track the ultrafast formation dynamics of size-selected hydronium clusters (H_2_O)*_n_*_−__1_H^+^, initiated from water clusters (H_2_O)*_n_* (*n* = 2–5). We observe a dramatic kinetic collapse in the reaction timescale, indicating that the onset of catalytic efficiency is abrupt rather than gradual. While the dimer (*n* = 2) exhibits a comparatively slow formation time of ∼259 fs, limited by a barrier to product separation, the addition of a single water molecule in the trimer (*n* = 3) accelerates this process to ∼70 fs, a rate that converges for larger sizes. *Ab initio* molecular dynamics (AIMD) simulations reveal that this acceleration is driven by the formation of a cyclic hydrogen-bond network that eliminates the reaction barrier, thereby facilitating the rapid separation of the hydronium ion from the hydroxyl radical. Further analysis of isomer-specific dynamics reveals this catalysis enhancement is strongly favored in the cyclic trimer configuration over the linear isomer.

## RESULTS AND DISCUSSION

### Experimental schemes

**Figure 1.**
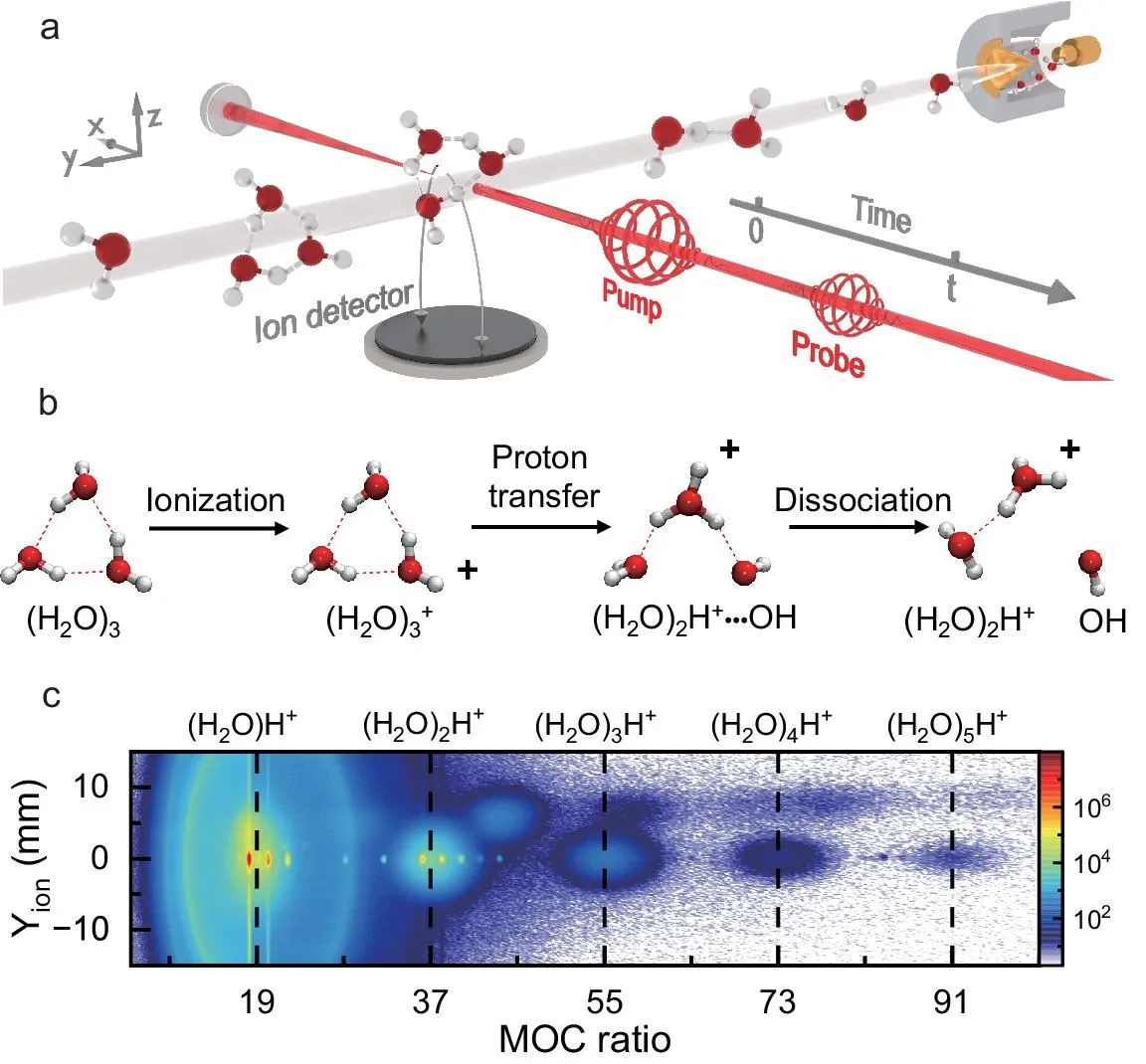
Experimental scheme. (a) Schematic illustration of the experimental configuration. (b) Overview of the light-induced formation mechanism of hydronium clusters (H_2_O)_3_H^+^. The plus sign symbolizes the ionized site. (c) Two-dimensional mass spectrum of the ion yields as a function of MOC and the detector’s *y*-axis impact position. The progression of hydronium clusters peaks, (H_2_O)*_n_*_−__1_H^+^ (*n* = 2–6), is highlighted by dashed lines.

The experimental measurements were performed in a reaction microscope based on cold target recoil-ion momentum spectroscopy (COLTRIMS) [26,27]. More details can be found in the ‘METHODS’ section. Briefly, as illustrated in Fig. 1a, water clusters were generated via the supersonic expansion of water vapor seeded in a neon carrier gas and subsequently interrogated by intense femtosecond laser pulses. The light-induced formation of hydronium clusters (H_2_O)*_n_*_−__1_H^+^ is initiated by the ionization of a neutral precursor (H_2_O)*_n_* and proceeds via a three-step mechanism, as depicted in Fig. 1b for water trimer (*n* = 3). First, an intense laser pulse singly ionizes a water molecule within the cluster via strong-field interaction, creating a highly acidic water cation H_2_O^+^ embedded within the solvent network. Second, driven by its instability, the H_2_O^+^ cation donates a proton to a neighboring water molecule, accompanied by ultrafast structural relaxation and the formation of a transient intermediate complex (H_2_O)*_n_*_−__1_H^+^···OH. Finally, this intermediate complex undergoes further structural relaxation and dissociation, allowing the hydronium cluster (H_2_O)*_n_*_−__1_H^+^ to emerge as a stable, free-flying ion, a step governed by the local structural dynamics of the cluster.

To track the (H_2_O)*_n_*_−__1_H^+^ formation dynamics in real time, we employed a disruptive probing scheme [25,28,29]. In this configuration, the initial intense pump pulse (1.5 × 10^14^ W/cm^2^) is followed by a time-delayed weak probe pulse (4 × 10^13^ W/cm^2^). Crucially, the probe intensity is kept sufficiently low to avoid ionizing neutral molecules. This ensures that the probe does not independently generate background ion signals from the neutral gas, but instead selectively interacts with the cationic species initiated by the pump. Acting as a disruptor, the probe pulse depletes the population of the evolving [(H_2_O)*_n_*_−__1_H^+^···OH] intermediates into alternative fragmentation channels if it arrives before the reaction is complete. By varying the delay between the pump and probe, we can map the timescale required for the system to relax into the stable product state. Both the pump and probe pulses are circularly polarized that drive the ejected photoelectron away from the parent ion and effectively suppressing electron recollision and recombination. Assisted by the electric field of the spectrometer, the released electron is rapidly swept away toward the electron detector located opposite to the ion detector, ensuring it does not interfere with the subsequent ionic relaxation. Furthermore, circular polarization minimizes ionization sensitivity related to molecular alignment. We note that while there is an extensive literature demonstrating that neutral water clusters can capture low-energy excess electrons to form dipole-bound anions or solvated electron states [30–33], such states do not participate in our observed dynamics. The high kinetic energy imparted to the photoelectron by strong-field ionization, combined with the continuous extraction field of the spectrometer, prevents the slow electron attachment required to stabilize such diffuse states. Consequently, our measurements exclusively isolate the dynamics of the cationic species.

### Identification of reaction products

Having established the experimental scheme, we first address the identification of the reaction products of interest. Figure 1c displays the ion yield distributions as a function of the detector’s *y*-axis impact position and the mass-over-charge (MOC) ratio. As indicated in the spectrum, a series of well-resolved isolated islands is observed, corresponding to hydronium clusters, (H_2_O)*_n_*_−__1_H^+^, derived from parent water clusters of varying sizes (*n* = 2–6). Accurate assignment of these daughter ions to specific neutral precursors is critical for the subsequent kinetic analysis. Previous studies have demonstrated that, for small water clusters, relaxation of the initially formed cation proceeds predominantly via ejection of a single hydroxyl radical (OH), while the fragmentation involving evaporative loss of neutral water molecules is negligible for clusters with *n* < 6 (decay fraction <10%) [34–36]. We therefore attribute the observed (H_2_O)*_n_*_−__1_H^+^ peaks primarily to the ionization of a specific parent (H_2_O)*_n_*^+^ precursors. This one-to-one correspondence enables us to utilize mass selection to isolate and analyze the reaction kinetics for individual cluster sizes [37]. Additional minor features in Fig. 1c arise from ionization of the carrier gas and background gas in the supersonic beam, with details provided in the Section S1.

### Formation time of (H_2_O)*_n_*_−__1_H^+^ ions

**Figure 2.**
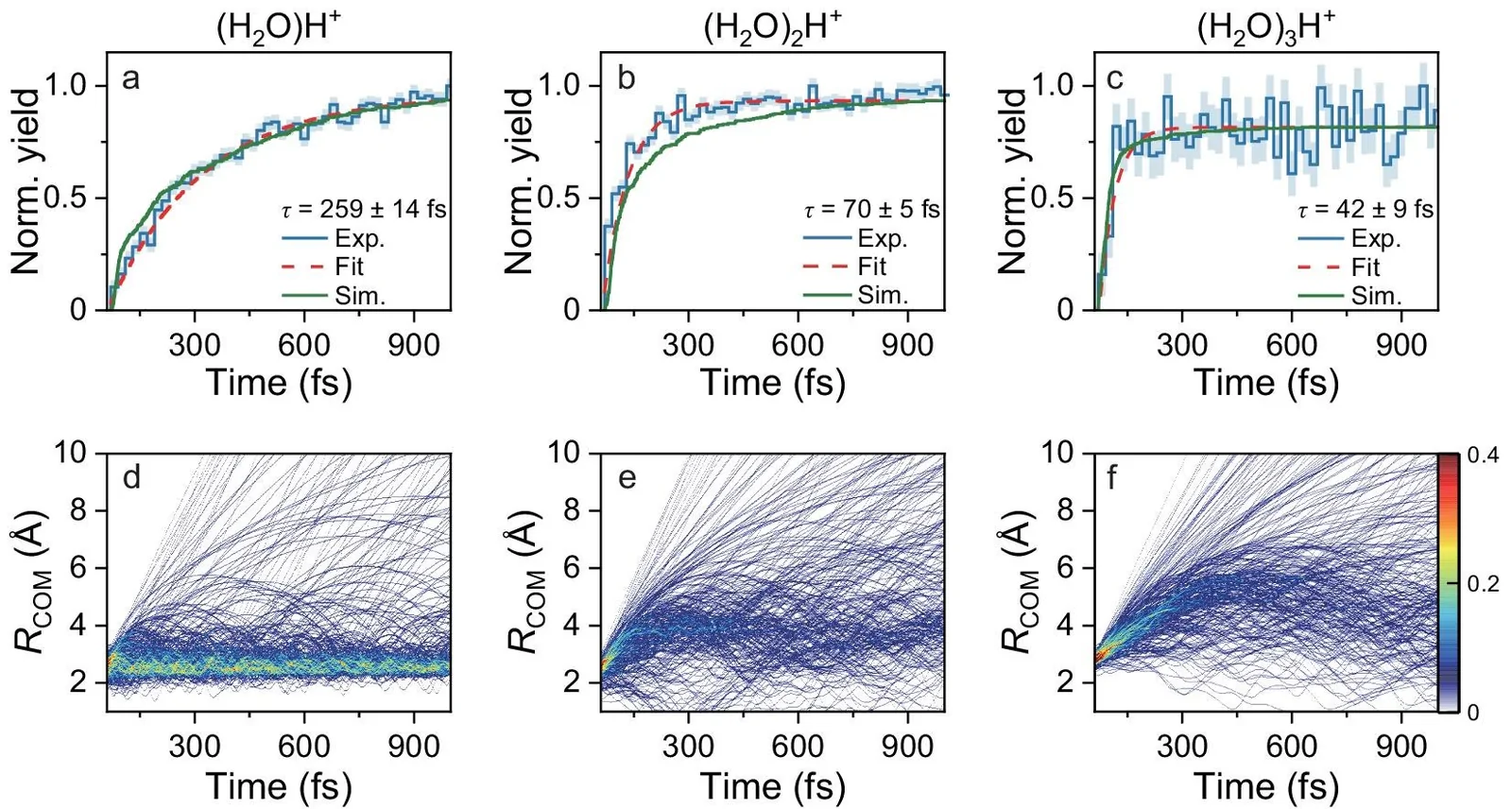
Size-dependent formation dynamics of hydronium clusters. (a–c) Normalized yields of (H_2_O)H^+^, (H_2_O)_2_H^+^ and (H_2_O)_3_H^+^ as a function of pump–probe time delay. Experimental results (blue) and simulation results (green) are shown alongside exponential fits to the experimental data (red-dashed curves). The yields were normalized to the [0, 1] interval after subtracting a time-independent background. The error bars represent statistical uncertainty, defined as $\sqrt {{N}_{{\mathrm{yield}}}} /{N}_{{\mathrm{norm}}}$, where *N*_yield_ is the raw fragment count at each time delay and *N*_norm_ is the normalization constant. (d–f) Simulated trajectories for the formation of (d) (H_2_O)H^+^, (e) (H_2_O)_2_H^+^, and (f) (H_2_O)_3_H^+^ fragments, respectively, tracking the center-of-mass distance (RCOM) between (H_2_O)*_n_*_−__1_H^+^ and OH species.

Figure 2a–c shows the normalized yields of (H_2_O)*_n_*_−__1_H^+^ (*n* = 2–4) fragments as a function of pump–probe time delay. For all cluster sizes, the ion signal exhibits a characteristic rise with increasing time delay before saturating at a constant plateau. This temporal profile directly tracks the progressive formation of stable hydronium clusters. At early times, the initially formed intermediate is depleted by the disruptive probe, resulting in a suppressed signal. As the delay increases, the system evolves toward the final product state, where the (H_2_O)*_n_*_−__1_H^+^ is effectively separated from the OH radical, rendering it stable against probe-induced depletion. The rising edge therefore maps the timescales of the formation process. Most notably, the formation of (H_2_O)H^+^ originating from (H_2_O)_2_ is significantly slower than that of larger clusters, providing direct experimental evidence of a unique kinetic barrier that impedes the dissociation of the hydronium–hydroxyl pair at this smallest cluster size.

**Figure 3.**
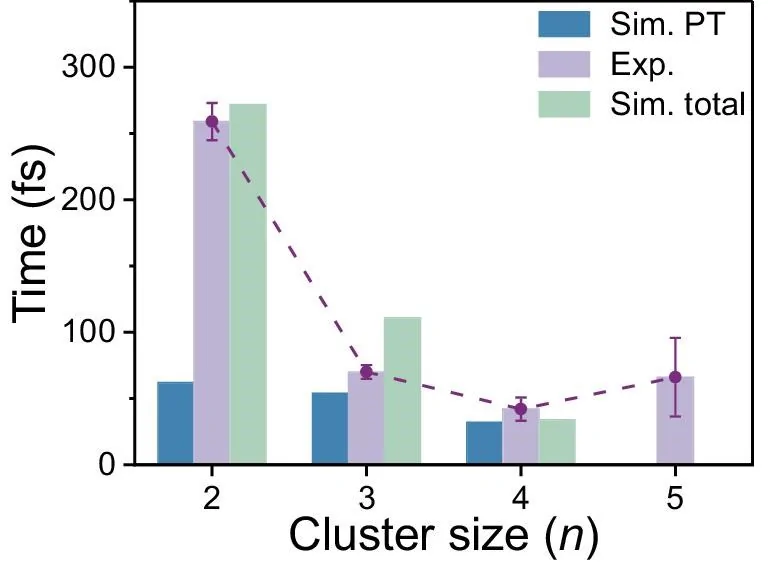
Summary of formation time across cluster sizes. Experimental formation times (purple dots) are extracted from the exponential fits in Fig. 2a–c (see Section S2 for (H_2_O)_4_H^+^). The error bars represent the fitting uncertainties. Simulation results display both the total formation time for the stable (H_2_O)*_n_*_−__1_H^+^ product (green bars) and the intrinsic proton transfer (PT) times (blue bars) for *n* = 2–4. Vertical colored bars are included to highlight the magnitude of the corresponding values.

To quantify the reaction kinetics as a function of cluster size, we modeled the delay-dependent ion yields using an exponential function, $Y( t ) = {y}_0 + {A}_0{\rm e}^{ - ( {t - {t}_0} )/{\mathrm{\tau }}}$, where *τ* represents the characteristic formation time constant. Here, *y*_0_ and *A*_0_ represent the baseline and amplitude, respectively. The onset time *t*_0_ was globally fixed at 65 fs, a value derived from our molecular dynamics simulations corresponding to the appearance time for 1% of trajectories, which showed negligible size dependence (<±10 fs). The experimentally extracted formation time constant *τ* is summarized in Fig. 3, revealing a dramatic decrease as the system transitions from the dimer to the trimer. The formation time drops from ∼259 ± 14 fs for (H_2_O)H^+^ to ∼70 ± 5 fs for (H_2_O)_2_H^+^, after which it converges for larger clusters. We note that although parent clusters up to *n* = 8 can be identified in our mass spectra, their number density drops precipitously in the molecular beam, precluding high-resolution pump–probe tracing. However, because the kinetic collapse and subsequent convergence are clearly resolved within the *n* ≤ 5 range, this regime comprehensively captures the critical transition to efficient catalysis. Crucially, the measured *τ* reflects the total duration required to form a stable (H_2_O)*_n_*_−__1_H^+^ product, a process comprising both the initial proton transfer and the subsequent separation and stabilization of the fragments. Since previous studies have established that the intrinsic proton transfer step occurs on an ultrafast, nearly size-independent timescale of ∼50 fs [22,38,39], the dramatic variation in *τ* reported here cannot be attributed to the proton transfer itself, but instead could arise from the product separation and stabilization stage. Therefore, the abrupt acceleration observed at *n* = 3 provides evidence that the water network with a larger size acts as a catalyst by accelerating the dissociation of the hydronium–hydroxyl pair.

### Molecular dynamics simulations: trajectory analysis

To elucidate the microscopic origin of the observed catalytic acceleration, we performed AIMD simulations (see ‘METHODS’ section for details). The calculations were initiated from the lowest-energy structures of (H_2_O)*_n_*(*n* = 2–4), evolving an ensemble of 300 nuclear trajectories for each size. In these trajectories, the formation of hydronium clusters is defined by *R*_COM_ > 3.2 Å (center-of-mass distance between hydronium clusters and hydroxyl radical), details are described in the ‘METHODS’ section and Section S3. As shown in Fig. 3, the results quantitatively reproduce the experimental results, confirming the dramatic collapse in reaction timescale. The simulated formation time drops from ∼272 fs for the dimer (experiment: ∼259 ± 14 fs) to ∼110.7 fs (experiment: ∼70 ± 5 fs) for the trimer, and further to ∼34 fs for the tetramer (experiment: ∼42 ± 9 fs). This quantitative agreement confirms that the simulation captures the essential physics of the reaction. Crucially, the results corroborate that the catalytic acceleration saturates beyond the trimer, identifying the addition of the third water molecule as the decisive structural switch that stabilizes the reaction efficiency.

Trajectory analysis reveals that this acceleration is driven primarily by the dynamics of product separation rather than the initial proton transfer. As shown in Fig. 3, the simulated intrinsic proton transfer times exhibit relatively modest variations across different cluster sizes: ∼62 fs for *n* = 2, ∼54 fs for *n* = 3 and ∼36 fs for *n* = 4 (see Section S4). This confirms that the significant variations in the total formation time *τ* are predominantly attributable to differences in the subsequent separation and stabilization dynamics. As visualized in Fig. 2d–f, a significant fraction of water dimers remains bound near the equilibrium distance after evolution, while larger clusters effectively generate separated products. The structural basis for this is depicted in Fig. 1b. In the trimer, the additional water molecule rotates its hydrogen atoms outward, destabilizing the hydrogen-bond network and enforcing an oxygen–oxygen face-to-face configuration. The resulting electrostatic repulsion drives rapid dissociation, a mechanism absents in the dimer. This enhanced formation efficiency is corroborated by the experimental mass spectra (Fig. S1), where stable non-dissociative parent ions (H_2_O)_2_^+^ are clearly captured for water dimer, whereas no distinct signal for non-dissociative parent cations (H_2_O)*_n_*^+^ is detected for clusters with *n* > 2. Thus, these results demonstrate that the water network acts as a catalyst, not only accelerating the reaction but also promoting efficient product separation.

### Molecular dynamics simulations: energy analysis

**Figure 4.**
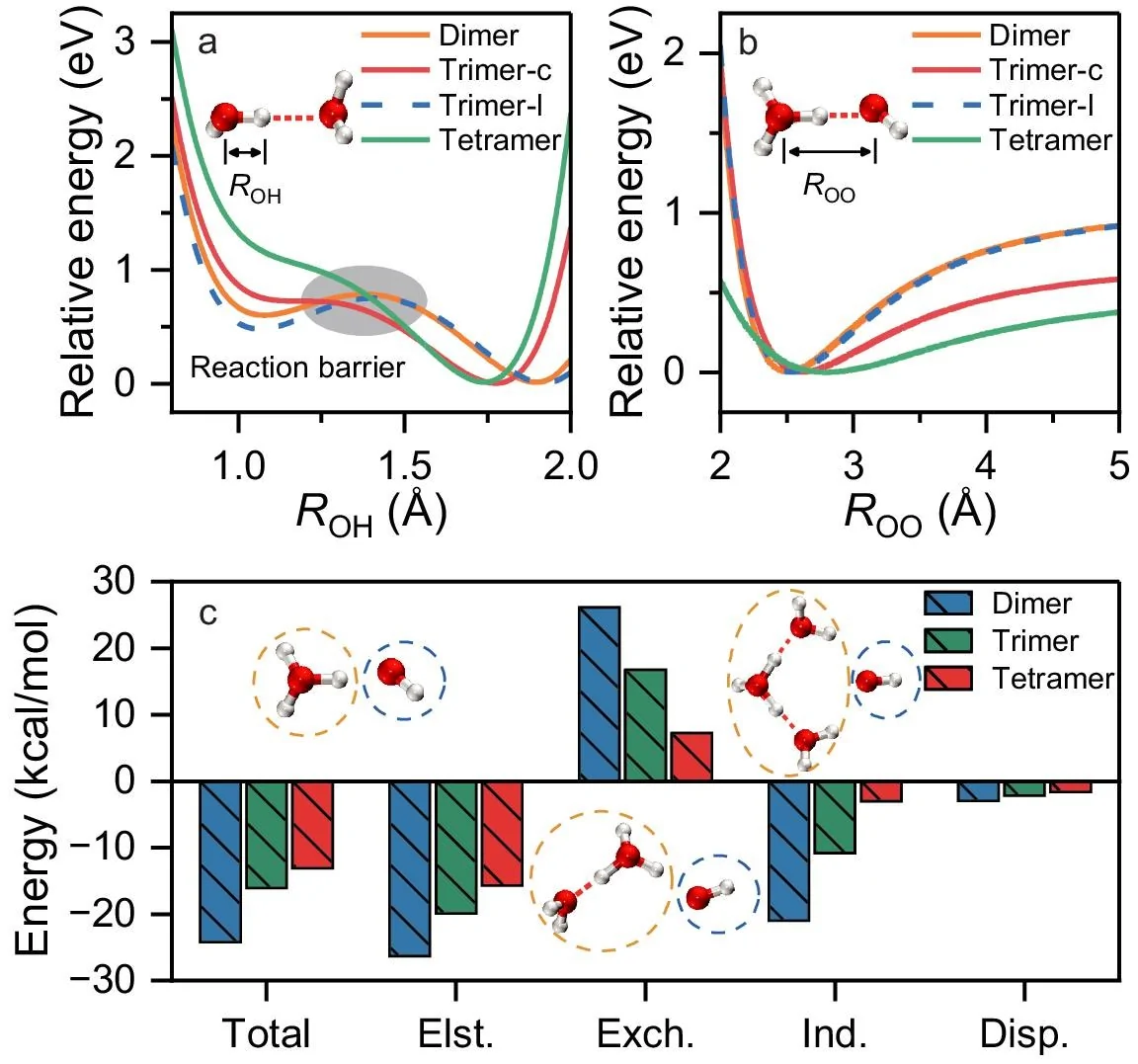
Calculated potential energy curves and interaction energy analysis. (a) Potential energy curves for proton transfer along the O–H coordinate (*R*_OH_) and (b) product separation along the O–O coordinate (*R*_OO_) for clusters (*n* = 2–4), computed at the DLPNO-CCSD(T)/aug-cc-pVTZ level. Note that the potential energy curves represent electronic energy profiles obtained from constrained scans to illustrate relative topographical changes across cluster sizes, rather than zero-point-corrected vibrationally adiabatic free-energy surfaces. The shaded area in panel (a) highlights the reaction barriers for water dimer or linear trimer. (c) SAPT decomposition of the interaction energy between the hydronium core (orange-dashed ellipse) and hydroxyl fragment (blue-dashed ellipse). Components shown include Elst., Ind., Disp. and Exch. terms. The insets display corresponding product structures for each cluster size.

Complementing the dynamical trajectory analysis, we performed high-level *ab initio* calculations to uncover the energetic origins of the catalysis dynamics. The potential energy surfaces (PES) for the reaction were computed using the DLPNO-CCSD(T)/aug-cc-pVTZ method. As shown in Fig. 4a, the potential energy profile along the hydrogen bond (*R*_OH_) reveals a noticeable barrier of 0.19 eV (4.38 kcal/mol) for the dimer cation, which effectively vanishes for the trimer and tetramer. Furthermore, as shown in Fig. 4b, the potential curves along the oxygen–oxygen bond (*R*_OO_), which governs the product separation, exhibit a significantly steeper barrier for the dimer compared to larger clusters. This energetic topology explains the tendency of the dimer to become kinetically trapped following proton transfer. Specifically, proton transfer is completed at short intermolecular distances, while the resulting nascent ion pair remains transiently trapped before subsequent structural evolution. This dynamic decoupling is further corroborated by a trajectory-based correlation analysis between the proton transfer coordinate and the O–O distance (see Section 6). The catalytic role of the additional water molecules is therefore rooted in their ability to eliminate the product-separation barrier present in the dimer, thereby reshaping the reaction energy landscape. By opening a favorable, low-barrier temporal window for the reaction, the extended hydrogen-bond network acts as an efficient catalyst that dramatically shortens the overall formation time of the hydronium ions.

To further identify the specific physical forces responsible for lowering these reaction barriers, we employed symmetry-adapted perturbation theory (SAPT). See Section S5 for more details. This method decomposes the total interaction energy between the OH fragment and the hydronium core into four distinct components: electrostatic (Elst.), induction (Ind.), dispersion (Disp.) and exchange (Exch.). The first three terms represent attractive forces (negative values), while the last term accounts for short-range repulsion (positive values). As shown in Fig. 4c, the total interaction energies weaken significantly as the cluster size increases, dropping from −24.2 kcal/mol for dimer to −16.1 kcal/mol for trimer, and further to −13.1 kcal/mol for tetramer. This substantial reduction in binding strength explains the lower dissociation barriers (Fig. 4b) and faster formation times (Fig. 3) observed for larger clusters. Notably, while the electrostatic forces typically dominate the attraction interaction, the much stronger interactions in the dimer are driven by a significant induction attraction contribution. This reflects the considerable mutual polarization effects between the separating fragments in the dimer, an effect that is effectively screened by the more delocalized solvent network in larger clusters.

### Isomer effect

**Figure 5.**
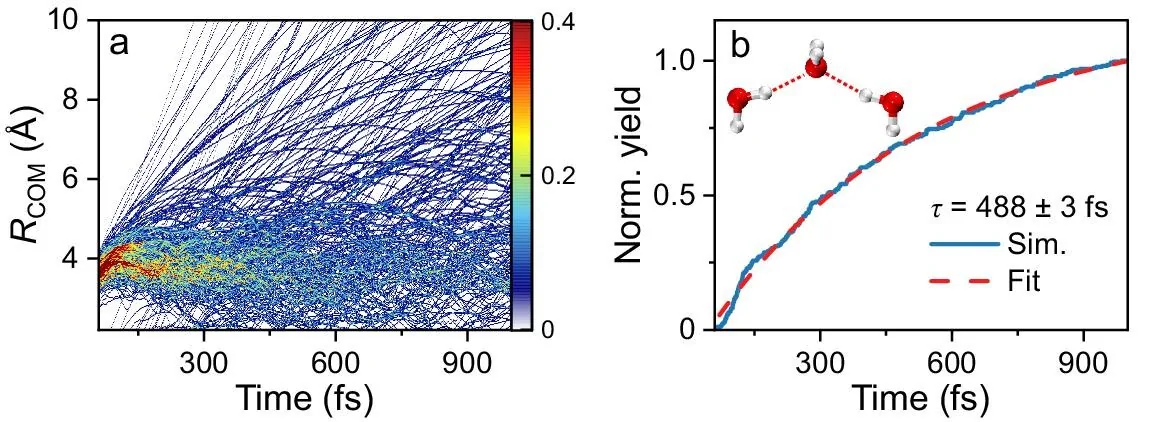
Isomer-dependent dynamics in the linear water trimer. (a) Simulated trajectories for the formation of hydronium fragment (H_2_O)_2_H^+^ for the linear water trimer configuration. *R*_COM_ represents the center-of-mass distance between (H_2_O)_2_H^+^ and OH species. (b) Corresponding simulated formation yields of (H_2_O)_2_H^+^. The simulation results and exponential fit are represented by blue and red curves, respectively. The molecular geometry of linear trimer is inserted in panel (b). A separation distance of 4.5 Å was adopted as the criterion for identifying the formation of (H_2_O)_2_H^+^ fragment, slightly exceeding the initial intermolecular separation of *R*_COM_ ∼ 4.0 Å in the linear trimer configuration.

To determine the structural sensitivity of this catalytic effect, we extended our AIMD simulations to examine the linear water trimer (trimer-l). Unlike the cyclic trimer (trimer-c), which represents the most stable geometry in our experiments, the linear isomer lacks cooperative ring-closure. Its open-chain structure prevents direct hydrogen-bond interaction between the terminal water molecules, and consequently, the reaction barrier persists. As displayed in Fig. 4a and b, the linear trimer exhibits a potential energy barrier of 0.28 eV (6.46 kcal/mol) along the reaction coordinates *R*_OH_ and a steep dissociation barrier along the *R*_OO_ coordinates, both of which are comparable to those of the dimer. This energetic landscape has profound kinetic consequences. As shown in Fig. 5(a), a large proportion of reaction trajectories remain kinetically trapped in a bound state near the equilibrium distance. For the subset of trajectories that do react, the extracted formation time for the linear trimer (trimer-l) is ∼488 fs (Fig. 5b), which is not only significantly slower than the cyclic trimer (trimer-c) but also slower than the water dimer. This result highlights that the mere addition of a third water molecule is insufficient to catalyze the reaction, rather, the catalytic effect is strictly structure-selective. It is the formation of a synergistic, three-body cyclic hydrogen-bond network that eliminates the reaction barrier. Thus, the joint experimental and theoretical results identify cyclic water trimer as the minimal structural unit required to switch on efficient catalysis in the hydronium clusters formation process. This sensitivity to local topology provides a microscopic foundation for condensed-phase observations. The importance of local topology observed here resonates conceptually with recent findings in condensed-phase environments. In bulk water and ice, structural ordering, such as quasi-one-dimensional wires [40] or lattice defects [41], strictly governs whether photogenerated ion pairs separate or recombine. Our findings reveal that this topological control in catalyzed formation of hydronium clusters emerges fundamentally with just three molecules.

## CONCLUSION

In conclusion, we have provided quantitative, real-time insight into the water-catalyzed formation of hydronium clusters (H_2_O)*_n_*_−__1_H^+^. By tracking the ultrafast dynamics across size-selected clusters from dimer to pentamer, we reveal a dramatic kinetic collapse in the formation timescale. The overall formation time collapses from ∼260 fs for the dimer to ∼70 fs for the trimer before converging for larger size, identifying the cyclic water trimer as the minimal functional unit required to switch on efficient catalytic dynamics. Our combined experimental and theoretical analysis demonstrates that this catalytic effect does not arise from accelerating the intrinsic proton transfer, but rather from eliminating the reaction barrier for the subsequent separation and stabilization of the ionic fragments through specific hydrogen-bonding networks. It is also worth noting that the stabilization of the separated ion pair in these small clusters may be further assisted by the amphiphilic nature of the nascent hydronium fragment. The surrounding vacuum could effectively act as a ubiquitous hydrophobic interface for the hydronium’s oxygen lone-pair face [42], creating an extreme local asymmetry that may engage the umbrella inversion mode and provide an additional mechanism for trapping the proton and further suppress recombination [43]. Furthermore, we show that this catalysis is exquisitely sensitive to topology, with the cyclic trimer configuration being vastly more effective than its linear isomer. Fundamentally, our results establish that water molecules act as structural switches, modifying the potential energy landscape to trigger efficient catalysis.

While this molecular-level insight provides a foundational framework for understanding ultrafast reaction dynamics in complex aqueous environments, we emphasize that gas-phase clusters inherently lack the extended solvation environment of the condensed phase. Although the minimal local topology governs the initial separation of the nascent hydronium–hydroxyl pair, subsequent long-range proton transport via the Grotthuss mechanism [12,13] instead relies on the dynamic tetrahedral hydrogen-bond network unique to bulk liquid water. We also note that the present AIMD simulations treat the nuclei classically to model the real-time heavy-atom structural reorganization. In reality, nuclear quantum effects (NQEs), such as zero-point energy and quantum tunneling, are known to significantly influence the structural and dynamical properties of protons in hydrogen-bonded networks by enhancing proton delocalization and further lowering effective reaction barriers [44,45]. Although explicitly incorporating real-time NQEs remains computationally beyond the scope of the present work, the classical treatment successfully captures the primary topology-driven elimination of the product-separation barrier that governs the observed kinetic collapse. In the actual quantum system, NQEs would likely act cooperatively with this topological structural switch, further facilitating the ultrafast charge separation process. Looking forward, extending this size-resolved approach to deuterated clusters (D_2_O) represents a promising future direction to uncover the role of kinetic isotope effects and NQEs in these fundamental water-catalyzed dynamics.

## METHODS

### Experimental setup

The experiments were performed using a Ti:sapphire multipass amplifier laser system, which delivers ultrashort pulses (25 fs, 790 nm) at a repetition rate of 10 kHz. The output laser beam was separated into pump and probe arms using a beam splitter with a 7:3 intensity ratio, and then recombined in a Mach–Zehnder-type interferometer. A quarter-wave plate was placed after combining two pulses to make them circularly polarized. The combined pulse sequence was focused onto the gas jet by a concave silver mirror (*f* = 7.5 cm) inside the COLTRIMS vacuum chamber. The time delay between the pump and probe pulses was controlled using a motorized delay stage. To prevent artifacts arising from optical interference (cross-correlation) between the pump and probe pulses, the exponential fitting of the ion yields was restricted to the delay range of 70–1000 fs.

Water clusters were produced via a supersonic expansion of a water vapor seeded in a neon carrier gas (4 bar driving pressure) through a 30-µm-diameter nozzle. The hydronium clusters (H_2_O)*_n_*_−__1_H^+^ created from the laser interaction were guided by a weak homogeneous electric field (7.1 V/cm) toward the ion detector of the COLTRIMS spectrometer. The three-dimensional momentum vectors of the resulting fragment ions were reconstructed based on their time of flight and impact position on the detector. The translational temperature of the jet was estimated to be ∼15 K using the relation *T*_trans_ = Δ*p^2^*/[4ln(4)*k*_B_*m*] [46–49], where *k*_B_ is the Boltzmann constant, and Δ*p* and *m* are the full width at half maximum of the momentum distribution in the jet direction (*y*-axis) and mass of the singly ionized monomers and dimers, respectively. Based on this low temperature, we assume that the water resides in its lowest-energy ground-state structures. Finally, we verified that the signal for the protonated dimer (H_2_O)H^+^ (m/q = 19) is not contaminated by non-dissociative water isotopologues (H_2_^17^O^+^ and HD^16^O^+^). Control measurements on the dominant water isotope H_2_^16^O^+^ (m/q = 18) showed no-delay-dependent modulation in ion yield. Consequently, any contribution from minor isotopes to the pump–probe signal at m/q = 19 can be ruled out.

### Simulation details

To elucidate the dynamics of hydronium formation, we performed AIMD simulations focused on the ground-state evolution of ionized water clusters following strong-field ionization. Under our experimental conditions (near-infrared laser pulses), ionization is dominated by the removal of an electron from the highest occupied molecular orbital (HOMO, 1b_1_), populating the ground ionic state. To quantify this selectivity, we calculated the vertical ionization energies for the HOMO, HOMO-1 and HOMO-2 orbitals at the IP-EOM-CCSD/aug-cc-pVTZ level [50,51], obtaining values of 11.72, 13.06 and 13.96 eV, respectively. Using the Ammosov–Delone–Krainov formula, the corresponding ionization rates are estimated to be 1.85 × 10⁻^6^, 2.56 × 10⁻^7^ and 6.35 × 10⁻^8^, respectively. This orders-of-magnitude difference confirms that ionization from the HOMO is overwhelmingly dominant. Therefore, our simulations exclusively model the dynamics on the ground ionic state PES. Because the ejected photoelectron rapidly leaves the interaction region, electron-ion recombination can be safely ignored, and the influence of excited states and nonadiabatic couplings is expected to be negligible under the present conditions. The equilibrium structures of neutral (H_2_O)*_n_* (*n* = 2–4) clusters were optimized at the MP2/aug-cc-pVDZ level [52] using the Gaussian 16 software package [53]. Vibrational frequencies calculations were performed at the same level to ensure the absence of imaginary frequencies. To simulate the proton transfer and dissociation dynamics following ionization, AIMD simulations were performed at the RI-MP2/aug-cc-pVDZ level using the ORCA 5.0.4 software [54,55]. The initial coordinates and velocities were obtained by a sampling of a quantum-harmonic Wigner distribution at a temperature of 50 K. Each trajectory was propagated for 1 ps with a time interval of 0.5 fs.

The formation of the hydronium cluster (H_2_O)*_n_*_−__1_H^+^ (*n* = 2–4) was monitored by tracking the separation between the reaction products. For each trajectory, the specific oxygen atom and its nearest hydrogen atom (determined by the O–H distance) that eventually form the hydroxyl radical (OH) fragment were identified. The remaining atoms were assigned to the (H_2_O)*_n_*_−__1_H^+^ fragment. The reaction progress was then quantified by calculating the center-of-mass distance (*R*_COM_) between these two moieties at every time step. A cutoff distance of *R*_COM_ > 3.2 Å was established as the robust criterion for the formation of a stable, separated (H_2_O)*_n_*_−__1_H^+^ product (see Supplementary data for details). This boundary effectively separates oscillatory bound trajectories from true dissociative trajectories, which allows us to capture the total duration of both the proton transfer and the subsequent separation dynamics in a single observable. We verified that while the choice of geometric descriptors or hyperparameters can influence absolute assignments [56], sensitivity testing using various *R*_COM_ cutoffs confirmed that the relative size-dependent kinetic trends reported here remain highly robust.

## Supplementary Material

nwag443_Supplemental_File

## References

[1] Douhal A, Lahmani F, Zewail AH. Proton-transfer reaction dynamics. Chem Phys 1996; 207: 477–98. 10.1016/0301-0104(96)00067-5

[2] Rosspeintner A, Lang B, Vauthey E. Ultrafast photochemistry in liquids. Annu Rev Phys Chem 2013; 64: 247–71. 10.1146/annurev-physchem-040412-110146 23298248

[3] Garrett BC, Dixon DA, Camaioni DM et al. Role of water in electron-initiated processes and radical chemistry: issues and scientific advances. Chem Rev 2005; 105: 355–90. 10.1021/cr030453x 15720157

[4] Ma J, Wang F, Mostafavi M. Ultrafast chemistry of water radical cation, H_2_O•^+^, in aqueous solutions. Molecules 2018; 23: 244. 10.3390/molecules23020244 PMC6017428 29373497

[5] Hammes-Schiffer S, Benkovic SJ. Relating protein motion to catalysis. Annu Rev Biochem 2006; 75: 519–41. 10.1146/annurev.biochem.75.103004.142800 16756501

[6] Garcia-Viloca M, Gao J, Karplus M et al. How enzymes work: analysis by modern rate theory and computer simulations. Science 2004; 303: 186–95. 10.1126/science.1088172 14716003

[7] Abou-Zied OK, Jimenez R, Romesberg FE. Tautomerization dynamics of a model base pair in DNA. J Am Chem Soc 2001; 123: 4613–4. 10.1021/ja003647s 11457253

[8] Kwon O-H, Zewail AH. Double proton transfer dynamics of model DNA base pairs in the condensed phase. Proc Natl Acad Sci USA 2007; 104: 8703–8. 10.1073/pnas.0702944104 PMC1885566 17502610

[9] Bogot A, Poline M, Ji MC et al. The mutual neutralization of hydronium and hydroxide. Science 2024; 383: 285–9. 10.1126/science.adk1950 38236956

[10] Geissler PL, Dellago C, Chandler D et al. Autoionization in liquid water. Science 2001; 291: 2121–4. 10.1126/science.1056991 11251111

[11] Moqadam M, Lervik A, Riccardi E et al. Local initiation conditions for water autoionization. Proc Natl Acad Sci USA 2018; 115: E4569–76. 10.1073/pnas.1714070115 PMC5960278 29712836

[12] Marx D, Tuckerman ME, Hutter J et al. The nature of the hydrated excess proton in water. Nature 1999; 397: 601–4. 10.1038/17579

[13] Agmon N . The Grotthuss mechanism. Chem Phys Lett 1995; 244: 456–62.

[14] Hassanali A, Giberti F, Cuny J et al. Proton transfer through the water gossamer. Proc Natl Acad Sci USA 2013; 110: 13723–8. 10.1073/pnas.1306642110 PMC3752248 23868853

[15] Headrick JM, Diken EG, Walters RS et al. Spectral signatures of hydrated proton vibrations in water clusters. Science 2005; 308: 1765–9. 10.1126/science.1113094 15961665

[16] Lin M, Singh N, Liang S et al. Imaging the short-lived hydroxyl-hydronium pair in ionized liquid water. Science 2021; 374: 92–5. 10.1126/science.abg3091 34591617

[17] Miyazaki M, Fujii A, Ebata T et al. Infrared spectroscopic evidence for protonated water clusters forming nanoscale cages. Science 2004; 304: 1134–7. 10.1126/science.1096037 15118121

[18] Shin JW, Hammer NI, Diken EG et al. Infrared signature of structures associated with the H^+^(H_2_O)*_n_* (*n* = 6 to 27) clusters. Science 2004; 304: 1137–40. 10.1126/science.1096466 15118122

[19] Wolke CT, Fournier JA, Dzugan LC et al. Spectroscopic snapshots of the proton-transfer mechanism in water. Science 2016; 354: 1131–5. 10.1126/science.aaf8425 27934761

[20] McDonald DC II, Wagner JP, McCoy AB et al. Near-infrared spectroscopy and anharmonic theory of protonated water clusters: higher elevations in the hydrogen bonding landscape. J Phys Chem Lett 2018; 9: 5664–71. 10.1021/acs.jpclett.8b02499 30205006

[21] Zhang C, Lu J, Feng T et al. Proton transfer dynamics following strong-field ionization of the water dimer. Phys Rev A 2019; 99: 053408. 10.1103/PhysRevA.99.053408

[22] Schnorr K, Belina M, Augustin S et al. Direct tracking of ultrafast proton transfer in water dimers. Sci Adv 2023; 9: eadg7864. 10.1126/sciadv.adg7864 PMC10337913 37436977

[23] van Harrevelt R, van Hemert MC. Photodissociation of water. I. Electronic structure calculations for the excited states. J Chem Phys 2000; 112: 5777–86.

[24] Stetina TF, Sun S, Lingerfelt DB et al. The role of excited-state proton relays in the photochemical dynamics of water nanodroplets. J Phys Chem Lett 2019; 10: 3694–8. 10.1021/acs.jpclett.9b01062 31091108

[25] Dantus M . Ultrafast studies of elusive chemical reactions in the gas phase. Science 2024; 385: eadk1833. 10.1126/science.adk1833 39116221

[26] Dörner R, Mergel V, Jagutzki O et al. Cold target recoil ion momentum spectroscopy: a ‘momentum microscope’ to view atomic collision dynamics. Phys Rep 2000; 330: 95–192.

[27] Ullrich J, Moshammer R, Dorn A et al. Recoil-ion and electron momentum spectroscopy: reaction-microscopes. Rep Prog Phys 2003; 66: 1463–545. 10.1088/0034-4885/66/9/203

[28] Jochim B, DeJesus L, Dantus M. Ultrafast disruptive probing: simultaneously keeping track of tens of reaction pathways. Rev Sci Instrum 2022; 93: 033003. 10.1063/5.0084837 35365005

[29] Dantus M . Tracking molecular fragmentation in electron-ionization mass spectrometry with ultrafast time resolution. Acc Chem Res 2024; 57: 845–54. 10.1021/acs.accounts.3c00713 PMC10956387 38366970

[30] Jordan KD, Wang F. Theory of dipole-bound anions. Annu Rev Phys Chem 2003; 54: 367–96. 10.1146/annurev.physchem.54.011002.103851 12626734

[31] Sommerfeld T, Jordan KD. Electron binding motifs of (H_2_O)*n*^−^ clusters. J Am Chem Soc 2006; 128: 5828–33. 10.1021/ja0587446 16637652

[32] Anusiewicz I, Skurski P, Simons J. Fate of dipole-bound anion states when hydrated. J Phys Chem A 2020; 124: 2064–76. 10.1021/acs.jpca.0c00360 32065750

[33] Clarke CJ, Michi Burrow E, Verlet JRR. The role of water molecules in the dissociation of an electron-molecule contact pair. Nat Commun 2025; 16: 2113. 10.1038/s41467-025-57403-7 PMC11876569 40032904

[34] Belau L, Wilson KR, Leone SR et al. Vacuum ultraviolet (VUV) photoionization of small water clusters. J Phys Chem A 2007; 111: 10075–83. 10.1021/jp075263v 17715907

[35] Dong F, Heinbuch S, Rocca JJ et al. Dynamics and fragmentation of van der Waals clusters: (H_2_O)*_n_*, (CH_3_OH)*_n_*, and (NH_3_)*_n_* upon ionization by a 26.5 eV soft X-ray laser. J Chem Phys 2006; 124: 224319. 10.1063/1.2202314 16784286

[36] Radi PP, Beaud P, Franzke D et al. Femtosecond photoionization of (H_2_O)*_n_* and (D_2_O)*_n_* clusters. J Chem Phys 1999; 111: 512–8. 10.1063/1.479330

[37] Hartweg S, Yoder BL, Garcia GA et al. Size-resolved photoelectron anisotropy of gas phase water clusters and predictions for liquid water. Phys Rev Lett 2017; 118: 103402. 10.1103/PhysRevLett.118.103402 28339280

[38] Tachikawa H, Takada T. Ionization dynamics of small water clusters: proton transfer rate. Chem Phys 2016; 475: 9–13. 10.1016/j.chemphys.2016.05.024

[39] Loh ZH, Doumy G, Arnold C et al. Observation of the fastest chemical processes in the radiolysis of water. Science 2020; 367: 179–82. 10.1126/science.aaz4740 31919219

[40] Tang F, Qiu DY, Wu X. Optical absorption spectroscopy probes water wire and its ordering in a hydrogen-bond network. Phys Rev X 2025; 15: 011048.

[41] Monti M, Jin Y, Mirón GD et al. Defects at play: shaping the photophysics and photochemistry of ice. Proc Natl Acad Sci USA 2025; 122: e2516805122. 10.1073/pnas.2516805122 PMC12663945 41264242

[42] Zhang P, Feng M, Xu X. Double-layer distribution of hydronium and hydroxide ions in the air–water interface. ACS Phys Chem Au 2024; 4: 336–46. 10.1021/acsphyschemau.3c00076 PMC11274287 39069983

[43] Hassanali AA, Giberti F, Sosso GC et al. The role of the umbrella inversion mode in proton diffusion. Chem Phys Lett 2014; 599: 133–8.

[44] Cassone G . Nuclear quantum effects largely influence molecular dissociation and proton transfer in liquid water under an electric field. J Phys Chem Lett 2020; 11: 8983–8. 10.1021/acs.jpclett.0c02581 33035059

[45] Waluk J . Nuclear quantum effects in proton or hydrogen transfer. J Phys Chem Lett 2024; 15: 598–607. 10.1021/acs.jpclett.3c03368 PMC10801683 38198616

[46] Wu J, Vredenborg A, Ulrich B et al. Nonadiabatic alignment of van der Waals–force-bound argon dimers by femtosecond laser pulses. Phys Rev A 2011; 83: 061403. 10.1103/PhysRevA.83.061403

[47] Litvinyuk IV, Lee KF, Dooley PW et al. Alignment-dependent strong field ionization of molecules. Phys Rev Lett 2003; 90: 233003. 10.1103/PhysRevLett.90.233003 12857255

[48] Dooley PW, Litvinyuk IV, Lee KF et al. Direct imaging of rotational wave-packet dynamics of diatomic molecules. Phys Rev A 2003; 68: 023406. 10.1103/PhysRevA.68.023406

[49] Hillenkamp M, Keinan S, Even U. Condensation limited cooling in supersonic expansions. J Chem Phys 2003; 118: 8699–705. 10.1063/1.1568331

[50] Kendall RA, Dunning TH Jr, Harrison RJ. Electron affinities of the first-row atoms revisited. Systematic basis sets and wave functions. J Chem Phys 1992; 96: 6796–806. 10.1063/1.462569

[51] Stanton JF, Gauss J. Analytic energy derivatives for ionized states described by the equation-of-motion coupled cluster method. J Chem Phys 1994; 101: 8938–44. 10.1063/1.468022

[52] Woon DE, Dunning TH. Gaussian basis sets for use in correlated molecular calculations. III. The atoms aluminum through argon. J Chem Phys 1993; 98: 1358–71. 10.1063/1.464303

[53] Frisch MJ, Trucks GW, Schlegel HB et al. Gaussian 16, Revision B.01. Wallingford, CT: Gaussian, Inc., 2016.

[54] Neese F, Wennmohs F, Becker U et al. The ORCA quantum chemistry program package. J Chem Phys 2020; 152: 224108. 10.1063/5.0004608 32534543

[55] Neese F . Software update: the ORCA program system—version 5.0. WIREs Comput Mol Sci 2022; 12: e1606. 10.1002/wcms.1606

[56] Di Pino S, Donkor ED, Sánchez VM et al. ZundEig: the structure of the proton in liquid water from unsupervised learning. J Phys Chem B 2023; 127: 9822–32. 10.1021/acs.jpcb.3c06078 37930954

