# Supplementary material for: Water-catalyzed ultrafast formation of hydronium clusters
Source: Natl Sci Rev. 2026 Jul 17;13(18):nwag443. doi: 10.1093/nsr/nwag443 (PMC13591639; doi:10.1093/nsr/nwag443)
Supplement: Supplementary file 1 [file nwag443_supplemental_file.pdf]

Supplementary Materials for

Water-catalyzed ultrafast formation of hydronium clusters

Menghang Shi[1,3], Chenxu Lu[1,3], Zhubin Hu[1,3], Wenbin Zhang[1*], Jiaxuan Chen[1], Peifen Lu[1], Haitao Sun[1†], Zhenrong Sun[1], Yang Tian[2‡] and Jian Wu[1§]

[1]*State Key Laboratory of Precision Spectroscopy, East China Normal University, Shanghai 200241, China*

[2]*Shanghai Key Laboratory of Green Chemistry and Chemical Processes, School of Chemistry and Molecular Engineering, East China Normal University, Shanghai 200241, China*

[3]*These authors contributed equally to this work*

[*] wbzhang@lps.ecnu.edu.cn

[†] htsun@phy.ecnu.edu.cn

[‡] ytian@chem.ecnu.edu.cn

[§] jwu@phy.ecnu.edu.cn

1. Time-of-flight (TOF) spectrum of ions

As discussed in the main text, the progression peaks corresponding to the ionic reaction products $(H_2O)_{n-1}H^+$ originating from parent $(H_2O)_n$ clusters can be clearly discriminated through mass selection. In addition to these dominant features, several minor peaks are observed in Fig. 1(c). As neon (Ne) gas was used as the carrier gas, these peaks primarily originate from ionization of Ne atoms (^{20}Ne) and its isotope ^{22}Ne. As marked in Fig. S1, the peaks at TOF ~ 4967.96 ns and ~ 5211.47 ns are predominantly assigned to $^{20}Ne^+$ and $^{22}Ne^+$, with mass-over-charge (m/q) of 20 and 22, respectively. The peak at ~ 6850 ns is primarily attributed to the mixed complex $(H_2O\text{-}^{20}Ne)^+$ (m/q = 38), while the peak at ~ 7025 ns mainly originates from $^{20}Ne_2^+$ (m/q = 40).

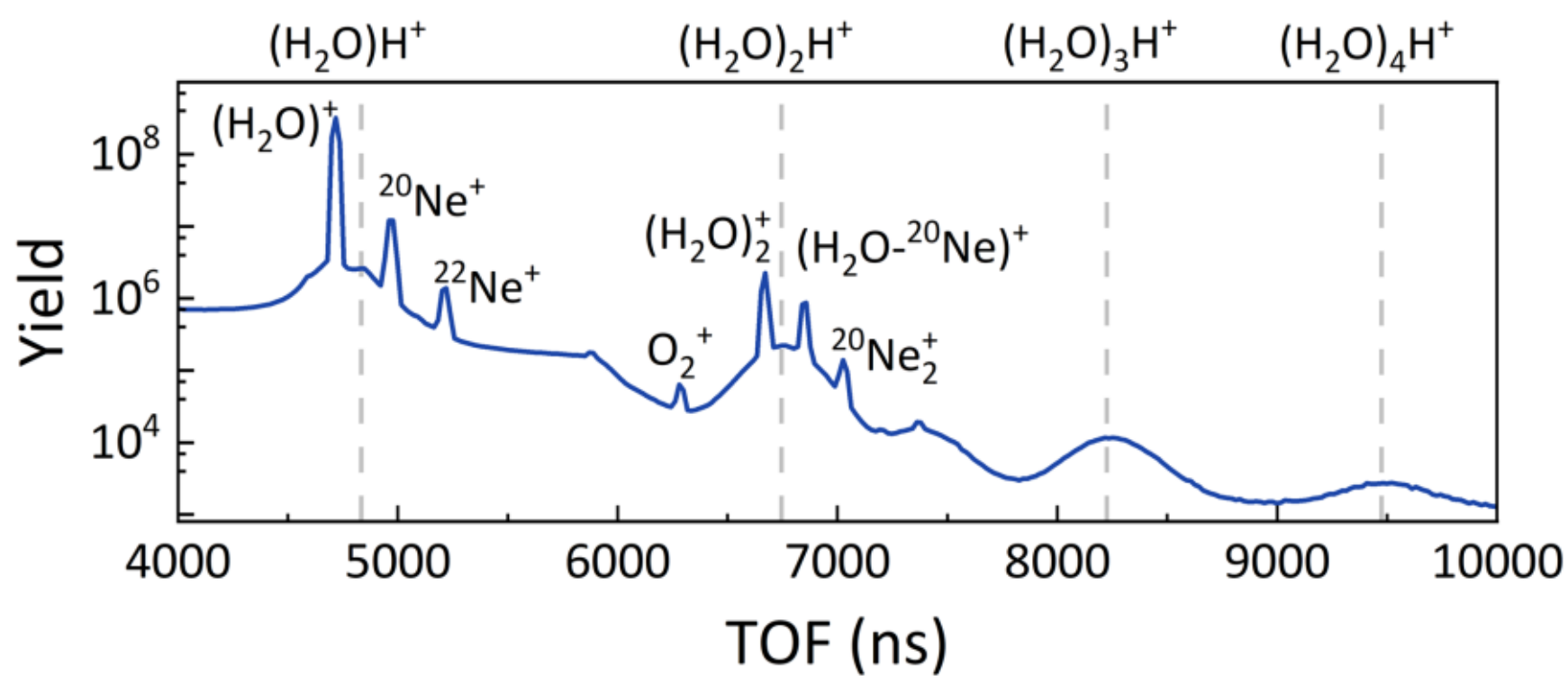

Figure S1. Measured TOF spectrum of ion fragments. The signals from different sizes of hydronium clusters, $(H_2O)_{n-1}H^+$ (n = 2 - 5), are marked with dashed lines.

2. Time-dependent yield of $(H_2O)_4H^+$ ions

Figure S2 shows the normalized yield of $(H_2O)_4H^+$ as a function of pump-probe delay. The fitting procedure and analysis are identical to those described in the main text. The extracted formation time constant, $\tau \sim 66 \pm 29$ fs, closely matches the formation times obtained for $(H_2O)_2H^+$ and $(H_2O)_3H^+$. These results are summarized in Fig. 3 of the main text. Despite the relatively large experimental uncertainties, the data exhibit a clear and rapid increase in yield with delay, confirming the ultrafast formation dynamics of the tetramer hydronium cluster.

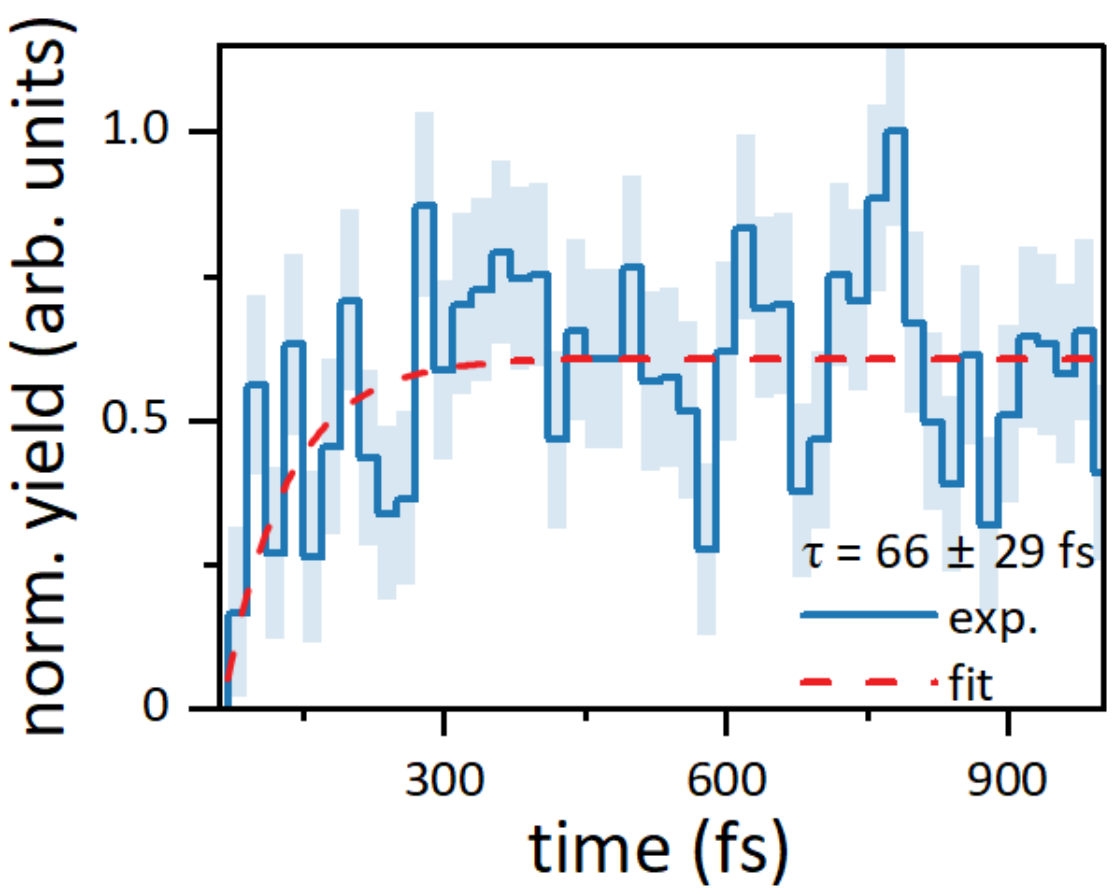

Figure S2. Normalized yield of $(H_2O)_4H^+$ as a function of pump-probe delay for water pentamer.

3. Criterion determination of the formation process

To compare with the experimental results, a criterion is needed to determine when the formation of stable hydronium clusters is completed in simulation. We use the center-of-mass separation between the hydronium cluster and hydroxyl radical, denoted as R_{COM}, as the metric for product separation. In our simulation, for each trajectory, the specific oxygen atom and its nearest hydrogen atom (determined by the O-H distance) that eventually form the hydroxyl radical (OH) fragment were identified. The remaining atoms were assigned to the $(H_2O)_{n-1}H^+$ fragment. Therefore, the reaction progress could be quantified by R_{COM} at every time step, as illustrated in Fig. S3(b). The simulated trajectories were propagated up to 1000 fs, matching the experimental observation time window, and analyzed by constructing a distribution of the center-of-mass distance between $(H_2O)H^+$ and OH fragments for each trajectory. As shown in Fig. S3, the main population of bound trajectories is clearly confined below a well-defined boundary at R_{COM} = 3.2 Å (red dashed line), indicating that separations smaller than this value correspond to non-dissociated configurations. This choice of threshold accurately reproduces the experimentally extracted formation time for the dimer (theory: 272 fs; experiment: 259 fs), demonstrating that it reliably captures the ultrafast formation dynamics in water dimer. By contrast, larger separations (e.g., R_{COM} > 3.5 Å) exceed the characteristic range of hydrogen-bonding interactions and are therefore not physically meaningful for defining bound intermediates [1]. For consistency, a uniform

distance criterion of R_{COM} = 3.2 Å is employed to define the formation of $(H_2O)_{n-1}H^+$ fragment across all cluster sizes in the simulations.

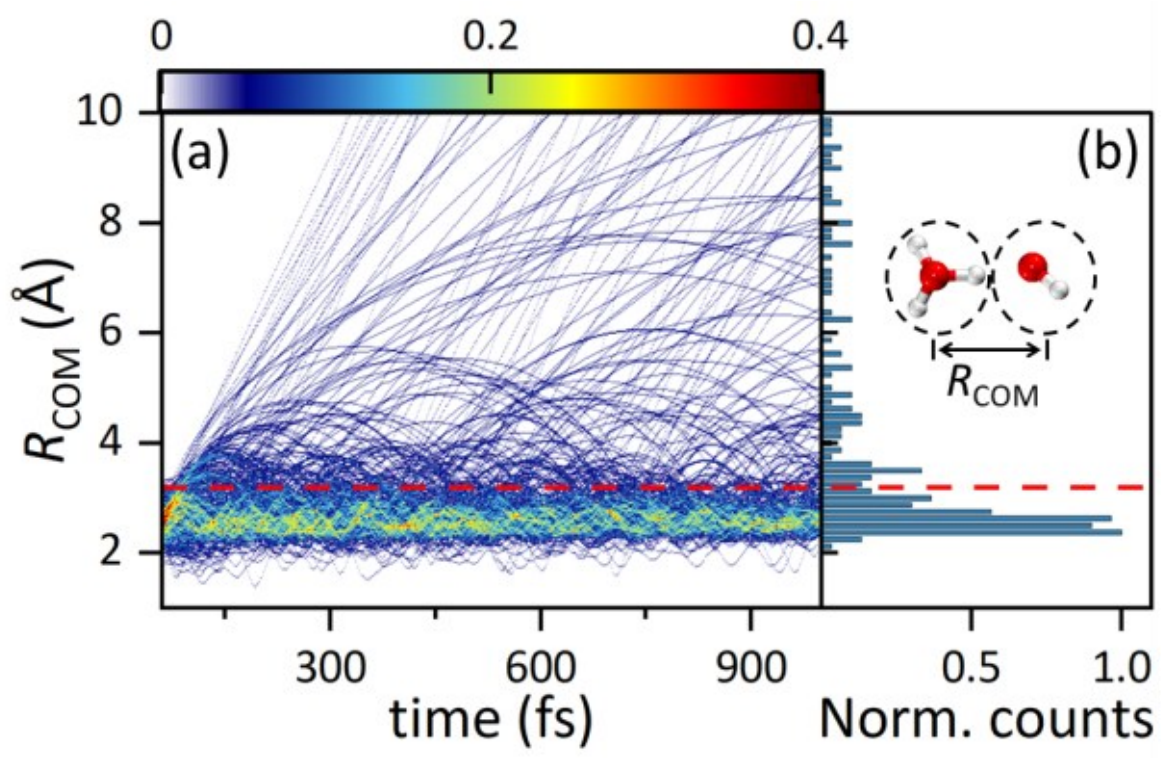

Figure S3. (a) Simulated trajectories for the separation (R_{COM}) of $(H_2O)H^+$ and OH fragment from water dimer after photoionization. (b) The R_{COM} distributions for trajectories at 1000 fs. The inset depicts the definition of R_{COM}.

4. Determination of proton transfer time

To quantify the proton transfer (PT) time following the ionization of water clusters $(H_2O)_n$ (n = 2 - 4), we analyzed the time-dependent population of ionic species across an ensemble of 300 independent *ab initio* molecular dynamics (AIMD) trajectories for each cluster size. For each trajectory, a successful PT event was defined by the simultaneous detection of a hydronium cation $(H_2O)_{n-1}H^+$ and a hydroxyl radical (OH) at a given time step. Structural identification was based on the following geometric criterion: the hydronium moiety was defined as an oxygen atom covalently bonded to three hydrogen atoms, while the hydroxyl radical was defined as an oxygen atom bonded to a single hydrogen atom. A covalent bond criterion of $d_{OH} < R_{cut}$ was applied, with the cutoff distance R_{cut} = 1.25 Å. This cutoff was determined based on the upper limit of stable O-H bond lengths observed following the initial relaxation phase. Then, at each time step, a binary status (1 if PT products are present, 0 otherwise) was assigned to each trajectory. The time-dependent probability of proton transfer (represented by the blue curves in Fig. S4) was obtained by summing the binary yields across the full ensemble of 300 trajectories. The characteristic PT time was extracted by fitting the time-dependent yield curves to an exponential function $y = y_0 + Ae^{-t/\tau}$, as indicated

by the red-dashed curves in Fig. S4), where τ represents the characteristic PT time. The fitted values for the dimer, trimer, and tetramer were determined to be 62 fs, 54 fs, and 36 fs, respectively.

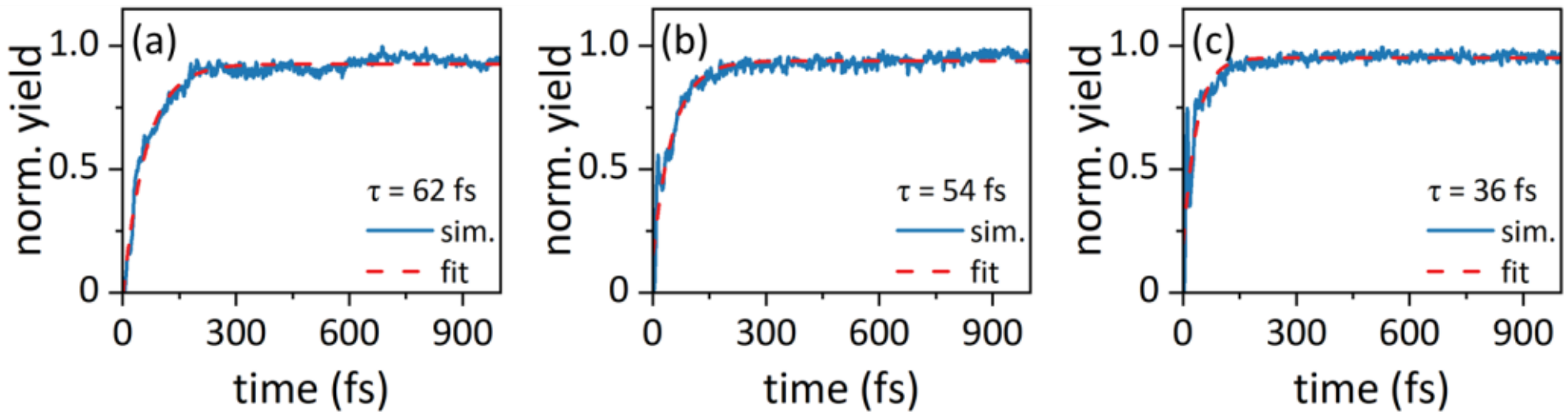

Figure S4. (a)-(c) Time-dependent yields and exponential fits of PT time for ionized water clusters $(H_2O)_n$ (n = 2 - 4). The yields were normalized to the [0, 1] interval after subtraction of a time-independent background. Blue curves represent the cumulative yields of the respective protonated species obtained from 300 independent AIMD trajectories. Red-dashed curves denote the first-order exponential fits of the form $y = y_0 + Ae^{-t/\tau}$.

5. Energy analysis of hydronium clusters

The potential energy surfaces of water cation clusters during the processes of proton transfer and dissociation were computed at the DLPNO-CCSD(T) [2]/aug-cc-pVTZ level with high accuracy. To reveal the nature of intra-/intermolecular interactions between $(H_2O)_{n-1}H^+$ and OH fragment, the energy decomposition analysis based on symmetry-adapted perturbation theory (SAPT) at the SAPT0 [3,4]/jun-cc-pVDZ [5] level for $(H_2O)_{n-1}H^+\cdot OH$ (n = 2 - 4) complex was conducted using PSI4 program[6]. SAPT is a first-principles approach based on perturbation theory that decomposes the total interaction energy between two molecular fragments into four fundamental components: 1) electrostatics (Elst.), describing the classical Coulomb interaction between charge distributions of the interacting molecules; 2) induction (Ind.) capturing the polarization and charge redistribution effects induced by the interaction partner; 3) dispersion (Disp.), accounting for attractive van der Waals forces arising from correlated electron fluctuations; 4) exchange (Exch.), representing short-range Pauli repulsion due to overlapping electronic wavefunctions. Below, we briefly outline

the theoretical framework of SAPT and describe its application in the present analysis.

For complex systems, the total Hamiltonian operator ($\hat{H}$) can be expressed as a sum of the Fock operator of the monomers ($\hat{F}$), the intermolecular perturbation operator ($\hat{V}$), and the intramolecular perturbation operator ($\hat{W}$):

$$\hat{H} = \hat{F} + \hat{V} + \hat{W} \tag{1}$$

The interaction energy between molecules or fragments can be expanded into an infinite series. The indices i and j represent the order of perturbation considered for $\hat{V}$ (intermolecular) and $\hat{W}$ (intramolecular), respectively:

$$\Delta E = \sum \Delta E^{(ij)} \tag{2}$$

Where i corresponds to intermolecular perturbations and j to intramolecular perturbations. Similar to the Møller–Plesset perturbation theory, the accuracy of SAPT increases as higher-order perturbation terms are included. Even the lowest-order SAPT approximation (SAPT0) provides interaction energy estimates significantly better than those obtained from Hartree-Fock (HF) calculations. The SAPT0, incorporates first- and second-order intermolecular perturbation effects. The interaction energy can be expressed as:

$$\Delta E_{\mathrm{SAPT0}} = \Delta E^{(10)}_{\mathrm{Elst.}} + \Delta E^{(10)}_{\mathrm{Exch.}} + \Delta E^{(20)}_{\mathrm{Ind.}} + \Delta E^{(20)}_{\mathrm{Exch_Ind}} + \Delta E^{(20)}_{\mathrm{Disp.}} + \Delta E^{(20)}_{\mathrm{Exch_Disp}} \tag{3}$$

where the terms Elst., Exch., Ind., and Disp., represent the electrostatic interaction, exchange repulsion, induction, and dispersion, respectively. The terms Exch_Ind and Exch_Disp represent coupling terms that mix exchange and induction/dispersion interactions. To simplify discussions, they are combined into the terms of induction or dispersion:

$$\Delta E_{\mathrm{Ind.}} = \Delta E^{(20)}_{\mathrm{Ind.}} + \Delta E^{(20)}_{\mathrm{Exch_Ind}} \tag{4}$$

$$\Delta E_{\mathrm{Disp.}} = \Delta E^{(20)}_{\mathrm{Disp.}} + \Delta E^{(20)}_{\mathrm{Exch_Disp}} \tag{5}$$

To improve the accuracy of the SAPT0 interaction energy, an additional δHF term is

introduced to account for higher-order corrections not considered in SAPT0:

$$\delta\text{HF} = \Delta E_{\text{HF}} - \left(\Delta E^{(10)}_{\text{Elst.}} + \Delta E^{(10)}_{\text{Exch.}} + \Delta E^{(20)}_{\text{Ind.}} + \Delta E^{(20)}_{\text{Exch}_{\text{I}}\text{nd}}\right) \quad (6)$$

where ΔE_{HF} represents the interaction, energy calculated using the HF method.

6. Dynamical correlation between proton transfer and product separation

To explicitly evaluate the dependence of the proton transfer coordinate (R_{PTC}) on the oxygen-oxygen separation distance (R_{OO}), we performed a trajectory-based R_{PTC}-R_{OO} analysis using the AIMD trajectories of the water dimer. This approach tracks the actual non-equilibrium dynamical pathways sampled after ionization, including thermal fluctuations, transient proton sharing, recrossing events, and product separation. It therefore provides a direct dynamical correlation between proton relocation and O-O separation under the same conditions used to extract the formation time.

For each trajectory, the initially attached oxygen atom of each hydrogen was identified from the first frame by the nearest-oxygen criterion. The final oxygen ownership was then determined from the majority assignment over the terminal portion of each trajectory, rather than from a single final frame. For a complete 1 ps trajectory, we used the last 100 fs; for shorter trajectories, we used only the last 20% of the available trajectory. This time-window criterion was used to avoid misclassification caused by instantaneous O-H vibrations or transient proton recrossing events. The 100 fs window spans multiple local vibrational periods and provides a stable estimate of the final proton localization, while the 20% cap prevents short trajectories from being dominated by an excessively long terminal window. The hydrogen atom whose oxygen ownership changed between the initial and final windows was assigned as the transferred proton, H_{transfer}. The donor oxygen, O_{donor}, was the oxygen to which H_{transfer} was initially attached, and the acceptor oxygen, O_{acceptor}, was the oxygen to which H_{transfer} was finally attached.

For every valid AIMD frame, we calculated $R_{\text{OO}} = r(O_{\text{donor}} - O_{\text{acceptor}})$ and $R_{\text{PTC}} = r(O_{\text{donor}} - H_{\text{transfer}}) - r(O_{\text{acceptor}} - H_{\text{transfer}})$. Under this definition, $R_{\text{PTC}} < 0$ corresponds to a proton localized on the donor side, $R_{\text{PTC}} = 0$ is the shared-proton boundary, and $R_{\text{PTC}} >$

0 denotes the transferred-proton (hydronium-like) region. Each trajectory was treated independently. Trajectories or trajectory segments that became structurally ambiguous or ran away were not extrapolated; only structurally valid frames with an unambiguous transferred proton were included in the R_{PTC}-R_{OO} correlation. In total, 234 trajectories contributed 433516 valid frames to this analysis, while no-transfer or ambiguous-transfer trajectories were excluded only from this correlation analysis.

As shown in Fig. S5, the binned mean R_{PTC} crosses zero at $R_{OO} \sim 2.09$ Å. After entering the positive- R_{PTC} region, a broad population remains at short-to-intermediate R_{OO} before separating to larger distances. This confirms that proton transfer occurs at relatively short O-O distances and can be completed while the hydronium/hydroxyl pair remains in close proximity, supporting the conclusion that the slow component in the dimer dynamics is associated with delayed product separation.

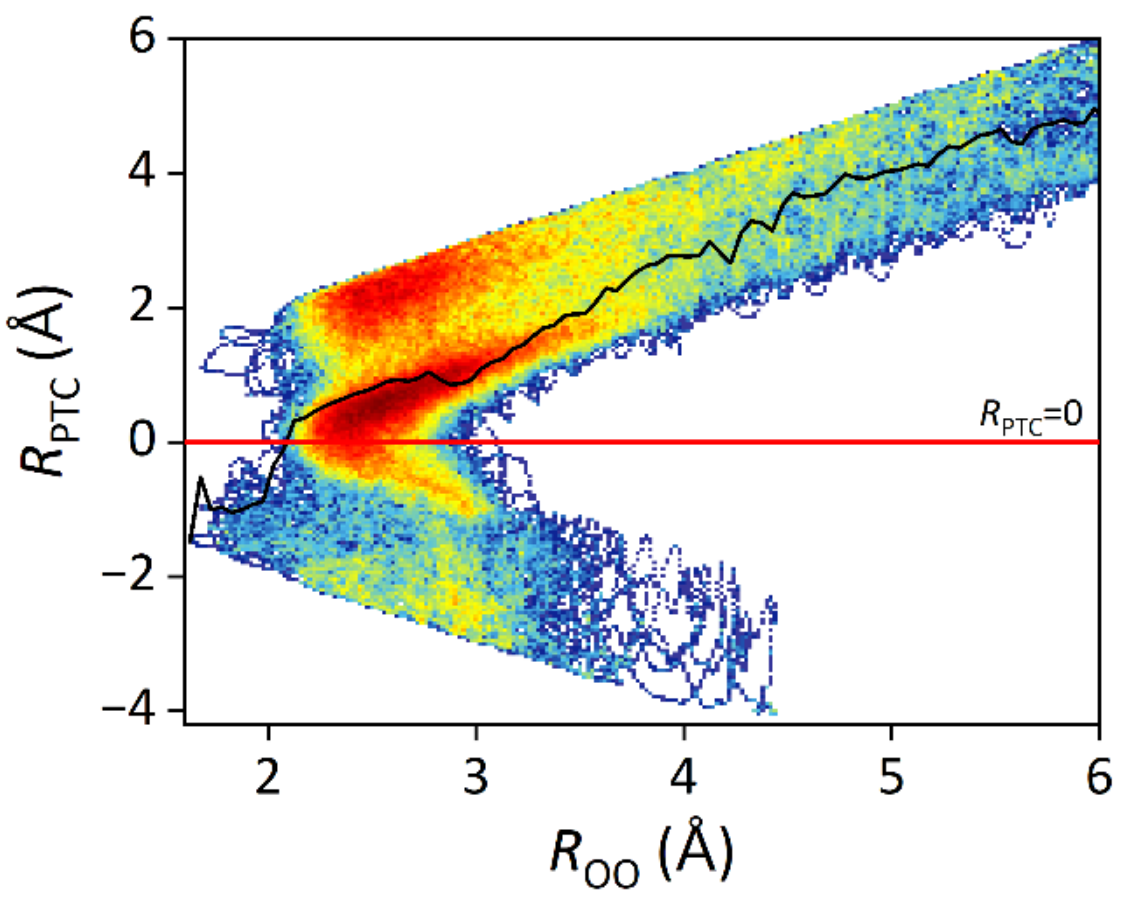

Figure S5. AIMD-derived correlation between the proton-transfer coordinate (R_{PTC}) and the donor-acceptor O-O distance (R_{OO}) for the water dimer. The red horizontal line marks $R_{PTC} = 0$, and the black curve shows the binned mean R_{PTC} as a function of R_{OO}.

Reference

[1] E. S. Brielle and I. T. Arkin, Quantitative Analysis of Multiplex H-Bonds, Journal of the American Chemical Society, **142**, 14150 (2020).

[2] C. Riplinger, P. Pinski, U. Becker *et al.*, Sparse maps-a systematic infrastructure

for reduced-scaling electronic structure methods. II. Linear scaling domain based pair natural orbital coupled cluster theory, J. Chem. Phys., **144**, 024109 (2016).

[3] E. G. Hohenstein and C. D. Sherrill, Density Fitting of Intramonomer Correlation Effects in Symmetry-Adapted Perturbation Theory, J. Chem. Phys., **133**, 014101 (2010).

[4] T. M. Parker, L. A. Burns, R. M. Parrish *et al.*, Levels of Symmetry Adapted Perturbation Theory (SAPT). I. Efficiency and Performance for Interaction Energies, J. Chem. Phys., **140**, 094106 (2014).

[5] E. Papajak, J. Zheng, X. Xu *et al.*, Perspectives on Basis Sets Beautiful: Seasonal Plantings of Diffuse Basis Functions, J. Chem. Theory Comput., **7**, 3027 (2011).

[6] R. M. Parrish, L. A. Burns, D. G. A. Smith *et al.*, Psi4 1.1: An Open-Source Electronic Structure Program Emphasizing Automation, Advanced Libraries, and Interoperability, J. Chem. Theory Comput., **13**, 3185 (2017).
